# Insights into familial Mediterranean fever: Chronic disease correlations with arthralgia and current health status of patients with familial Mediterranean fever in Jordan

**DOI:** 10.1515/rir-2025-0003

**Published:** 2025-04-02

**Authors:** Mai I. Al-Hawamdeh, Farah Othman, Safaa’ Taha, Tityana Adawı, Talal Aburjaı

**Affiliations:** Department of pharmacy, College of pharmacy, Amman Arab University, Amman, Jordan; Department of Pharmaceutical Sciences, Faculty of Pharmacy, The University Of Jordan, Amman, Jordan

**Keywords:** familial mediterranean fever, familial mediterranean fever, Jordan • premarital screening

## Abstract

**Background and Objectives:**

Familial Mediterranean fever (FMF) stands as a significant challenge within Jordan’s clinical practice, despite its low prevalence of 0.04% within the Jordanian population. This study aims to investigate the current status of the health status of FMF patients in Jordan while exploring any present associations between chronic diseases and the severity of their symptoms.

**Methods:**

This is a cross-sectional descriptive survey-based study conducted during the period between 1st of March till the last of May 2023 in Jordan. The survey was distributed randomly to a group of FMF patients, Sample size was based on FMF prevalence in Jordan (0.04%); study sample (*N* = 67) included FMF patients in Jordan from different age groups. All results were performed through proper statistical analysis.

**Results:**

The study includes 67 FMF patients, predominantly Jordanian and aged 18–31, revealed that 58.2% only were diagnosed through blood genetic testing. Marriages among first-degree relatives showed a 60% probability of FMF transmission compared to 10% in non-related parents (*P* = 0.001), leading 82.1% of participants to call for pre-marital testing. Acute symptoms included abdominal pain, fever, arthralgia, and myalgia, with hypertension being the most frequent comorbidity (14.9%) and significantly associated with myalgia and arthralgia (*P* < 0.05). Colchicine was the primary treatment for 89.6% of patients, with high adherence rates (90.3%).

**Conclusion:**

Among chronic comorbidities, hypertension was associated in increasing the severity of the myalgia during attacks. The issue of misdiagnosis remains a major challenge in Jordanian clinical practice. Our findings assert the importance of future incorporation of FMF premarital testing.

## Introduction

Familial Mediterranean fever (FMF) is one of the world’s most common inherited auto-inflammatory diseases. Even though it mainly affects populations from the East Mediterranean region, FMF cases are reported worldwide. According to past research, the prevalence of FMF in Jordan has been estimated to be 0.04%.^[1]^ FMF is caused by functional mutations in the MEFV gene, which codes a protein called pyrin that mainly regulates the intrinsic immune system.^[2]^ FMF symptoms are described by self-limiting events of fever associated with arthritis, serositis, and dermal manifestations, lasting 12–72 h with a variable recurrence rate.^[3]^

Recent systematic studies have defined several inflammatory conditions in FMF, like arthritis, rash, peritonitis, pleuritis, and typically fever.^[4, 5, 6]^ FMF unveils a complex interplay with chronic diseases such as hypertension, shedding light on the multifaceted health challenges faced by patients. Recent studies have underscored a significant association between FMF and hypertension, implicating potential synergistic effects on symptom severity.^[7]^ Since diagnosis of FMF is challenging in clinical practice, several arrays of classification and diagnostic standards for FMF have been used. The first criteria was set for adults by the specialists in Hashomer Medical Centre in 1997,^[8,9]^ followed be criteria’s implemented for children diagnosis purposes in 2009.^[10]^ Colchicine has been the primary treatment for FMF since 1972.^[11]^ Colchicine is correlated to pyrin and acts by changing the organization of actin cytoskeleton through binding to tubulin monomers and preventing polymer formation.^[12]^

Based on the current research, it is highly recommended to assess genetic analysis of the MEFV gene in order to clearly diagnose a patient as having FMF, however diagnosis based on clinical presentation is still widely implemented in Jordan.^[13]^ Few studies investigated the status of FMF in Jordan, with their main focus was on children with FMF and finding FMF mutation types in Jordan.^[14, 15, 16]^

Status of the general health of FMF patients at different age groups was not investigated recently in Jordan, neither any association of the disease status with several important factors like the degree of kinship between parents and spouses, treatment compliance, patient demographics, and pregnancy status. Our study aims to update the current health status of FMF patients in Jordan from patient point of view, to find any present association between chronic diseases and severity of symptoms, and to assess if there is a need for FMF premarital testing in Jordan.

## Methods

### Study Design and Participants

This is a cross-sectional descriptive survey-based study carried during the period between 1st of March till the last of May 2023 in Jordan to assess general health status of FMF patients in Jordan and to investigate the need for premarital testing for FMF in Jordan. The study targeted population resident in Jordan who are Jordanian or non-Jordanian and were diagnosed by medical specialist with FMF.

This study used a self-administered survey containing multiple choice questions that was designed and distributed using Google forms. The survey was written and distributed in Arabic language with medical terminology being simplified to the public using public terms. Participants were given clarification on ambiguous words such degree of kinship; first degree relatives were defined as first cousins from the mother’s or father’s side, whilst second degree relatives were defined as other relatives.

### Study Instrument

The survey was constructed using Google forms; it included mandatory twenty-eight multiple choice items, twelve yes/no items, table 1. Initial draft of the survey was performed based on deep literature review.^[13,17,18]^ Face and content validity was done by three medical doctors specialized in FMF and six experts in the field through evaluating the relevancy, clarity, spelling and comprehension of different parts of the survey. Internal reliability was assessed by Cronbach’s alpha coefficient (α = 0.72). The survey was designed to take less than five minutes for participants to be completed.

The final version of the survey included five main sections. First section was related to demographic data including gender, age, nationality, birth place, residency place, and the degree of kinship between parents, marital status, and the degree of kinship between spouses. Second section was about patient medical history including diseases status, Diagnosis method, family history, age of disease onset, chronic diseases. Third section investigate patient current health status like current present symptoms like abdominal pain, fever, arthritis, muscle pain, general fatigue or insomnia. The fourth one was related to patients’ medications including name of received medication, patient administration and compliance. Finally, the last section was about female patients’ pregnancy and delivery history including previous pregnancy history, treatment during pregnancy, any present preterm delivery, any present apportion, any present children’s disability and any present children’s with FMF.

### Study Sample and Survey Application

Study sample included patients diagnosed with FMF and resident in Jordan. The study survey was prepared using Google form and distributed by web through different social platforms like Facebook (FMF patients group) and through mobile massages applications like WhatsApp.

Each participant was allowed to send one response besides answering all the questions of the survey that were mandatory; so that the participant would not be transferred to the next question unless he/she completed answering the previous one. Data collection was monitored by the research team during the sampling period and peer reviewed to ensure proper filling of the survey and measure reliability and avoid duplication of responses.

### Sample Size

Sample size calculation was based on published literature and convenience sample size design that was used based on the below mentioned equation.^[19, 20, 21]^ A sample size of minimum fifty-nine patients will be needed as follows:

N = [(z α/2) 2* P*(1-P)] / d2;^[19]^

*Z α/2 = 1.96, based on type 1 error of 0.05*;

*P = 0.04, where p is the prevalence from previous studies*;*^[22]^*

*d = 0.05, and d is the precision of the study and considered 0.05 for good precision*.

**Table 1:** survey questions and options

Questions	Options
1. Are you willing to participate in this study?	- Agree
	- Disagree
2. What is your nationality?	- Jordanian
	- Non-Jordanian
3. What is your age?	- Under 18 years
	- 18-30 years
	- 31-40 years
	- 41-50 years
	- Over 50 years
4. Place of birth?	- Jordan
	- A country in the Mediterranean region
	- Other
5. Place of residence?	- A governorate from the central region (Amman, Madaba, Zarqa, Salt, Zarqa)
	- A governorate from the northern region (Irbid, Mafraq, Jerash, Ajloun)
	- A governorate from the southern region (Karak, Tafileh, Ma’an, Aqaba)
6. What is the degree of kinship be- tween your father and mother?	- First-degree relatives (cousins)
	- Distant relatives
	- No kinship between father and mother
7. Marital status?	- Married
	- Not married
8. What is the degree of kinship with your spouse?	- First-degree relatives (cousins)
	- Distant relatives
	- No kinship
9. Do you believe that it is necessary to conduct a Mediterranean Fever test	- Yes
before marriage?	- No
10. Are you affected by or a carrier of Mediterranean Fever?	- I have the disease
	- I am a carrier of the disease
11. How was your Mediterranean Fever diagnosis confirmed?	- By conducting a specific blood test
	- Suspected appendicitis or gallbladder infection
	- After appendix removal, the disease was discovered
	- Based on symptoms only
	- Other
12. Does anyone in your family suffer from Mediterranean Fever?	- One of them
	- All of them
	- Parents
	- Siblings
	- Children
	- Maternal uncles/aunts
	- Paternal uncles/aunts
13. When did you first experience symp- toms of Mediterranean Fever?	- Since birth
	- Between ages 5-12
	- Between ages 13-20
	- Age 21 or older
14. Do you suffer from any other chronic diseases?	- Hypertension
	- Diabetes
	- Asthma
	- Digestive system diseases
	- Autoimmune diseases
	- Cardiovascular diseases
	- Urinary system diseases
15. Do you suffer from severe pain at- tacks in the abdomen?	- Yes
	- No
16. Do you experience fever attacks?	- Yes
	- No
17. Do you suffer from joint pain or swelling attacks?	- Yes
	- No
18. Do you experience muscle pain attacks?	- Yes
	- No
19. Are your attacks linked to insomnia, fatigue, or stress?	- Yes
	- No
20. Do you feel completely healthy after the attacks?	- Yes
	- No
21. What treatment are you receiving?	- Colchicine
	- Biological medication
	- Cortisone
	- Other
22. Are you committed to taking the treatment prescribed by the doctor?	- Yes
	- No
23. Do you take the medication regu- larly or only during an attack?	- Regularly
	- During an attack or when symptoms worsen
24. Do you take other medications be- sides Colchicine to alleviate the attacks?	- Yes
	- No

The study data collection period was performed within two months. During this period 67 subjects completed the survey.

### Ethical Consideration

The study was reviewed and approved by Institutional Review Board (IRP) at University of Jordan, Deanship of Scientific Research (IRP reference: 132/2023). Informed consent was included as the first part of the survey and had been obtained from each participant prior to participation. No data were saved before the participants submitted their complete answers, and the participants were free to leave the study at any time without providing a reason.

### Statistical Analysis

Statistical tests were performed using the Statistical Package for the Social Sciences (SPSS) version 27.0 (SPSS Inc. Chicago, IL, USA, 2020). Initially, descriptive statistics were conducted for demographic variables, medical history data, current disease status, pregnancy, and delivery history. All data was represented by frequencies and percentages. The association of various side effects and chronic disease was assessed using the chi-squared test (χ^2^), with a confidence level of 95% and significance value *P* ≤ 0.05.

## Results

A total of 67 patients diagnosed with FMF accepted to participate in the study and successfully completed the survey. The majority of participants were Jordanian (*n* = 55, 82.1%), between 18–31 years old (*n* = 22, 32.8%), born in Jordan (*n* = 51, 76.1%) and resident in the central region (*n* = 30, 44.8%) or the northern part of Jordan (*n* = 34, 50.7%). Of all the participants 82% were identified as FMF patients while 18% were identified as FMF gene carriers. Detailed demographic data are shown in Table 2.

**Table 2:** Demographic Data of the study sample

Parameter	Frequency (%)
Nationality	
Jordanian	55 (82.1)
Non-Jordanian	12 (17.9)
Gender	
Female	35 (52.3)
Male	32 (47.7)
AGE (years)	
≤ 18	16 (23.9)
18-30	22 (32.8)
31-40	15 (22.4)
41-50	8 (11.9)
≥ 51	6 (9.0)
Region of Residency	
Middle Jordan	30 (44.8)
North Jordan	34 (50.7)
South Jordan	3 (4.5)
Birthplace	
Jordan	51 (76.1)
Arab Mediterranean countries	11 (16.4)
Other	5 (7.5)
Marital Status	
Married	39 (58.2)
Single	28 (41.8)
Disease Status	
FMF patient	55 (82)
FMF Carrier	12 (18)

Out of the study subjects 7.5% of the married FMF patients were first degree relatives; cousins from mother side, father side or both, while 7.5% were second degree relatives and 73.1% were not. However according to our results first degree related parents diagnosed with FMF revealed the probability of 60% of transmitting FMF to their offspring’s. In comparison with non-related parents diagnosed with FMF only 10% probability exist (Chi-square test; 2, *N* = 67, χ^2^ = 13.58, *P* = 0.001,). With regard to the kinship between FMF patient parents, 25.4% (*n* = 17) were first degree relatives, while 31.3% (*n* = 21) were second degree relatives. Therefore, 82.1% (*n* = 45) of the study patients believe that is important to perform pre-marital testing for FMF mutations.

Regarding FMF diagnosis, blood genetic testing is considered the first practice (*n* = 39, 58.2%). However, 23.9% of the patients were still diagnosed based on symptoms, 3% based on appendix removal then blood testing, 6% based on suspected appendicitis while the rest were diagnosed by chance. Most of the patients start to suffer from FMF symptoms between the age 5–12 years (*n* = 24, 35.8%) or 13–20 years (*n* = 21, 31.3%). On the other hand, a minority of the participants experienced their first symptoms before 5 years or after 21 years (9% and 13.5% respectively).

It is noteworthy to mention that most of the patients suffer from episodes of abdominal pain, fever, arthralgia and myalgia during acute disease attack. Moreover, most patients suffer from insomnia and general fatigue post attack. Table 3 shows the relative frequencies and percentages to such symptoms. Among the patients, only 75.4% feel well post FMF attack.

**Table 3:** Current health status among study FMF patients during and post disease attack

Symptoms	Yes, Frequency (%)	No, Frequency (%)
During Disease Attack (*n* = 65)		
Episodes of abdominal pain	50 (76.9)	15 (22.4)
Episodes of Fever	44 (67.7)	21 (32.3)
Episodes of Arthralgia	37 (56.9)	28 (43.1)
Episodes of Myalgia	42 (64.4)	23 (35.4)
Post Disease Attack (*n* = 63)		
Insomnia	42 (66.7)	21 (33.3)
General fatigue	42 (66.7)	21 (33.3)
General Health status (*n* = 65)		
Optimum general health	49 (75.4)	16 (24.6)

**Figure 1:**
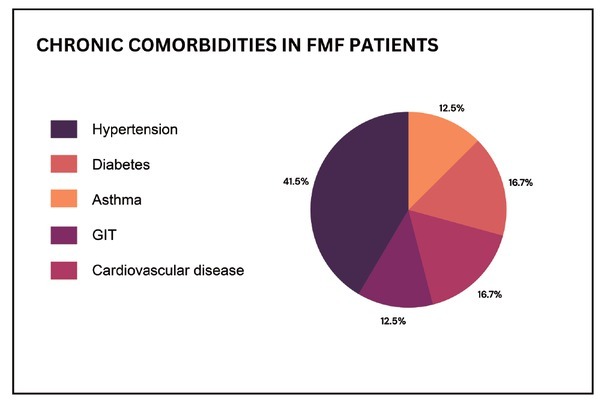
FMF patients with chronic comorbidities (N =24 out of 67). GIT, gastrointestinal tract.

According to the study results, the participants suffer from other chronic comorbidities like diabetes, hypertension, asthma, gastrointestinal tract diseases, autoimmune diseases, cardiovascular diseases, or urinary tract diseases. As shown from Figure 1, hypertension was the most common incident among study participants. Using Chi-square test, there was an association between hypertension among FMF patients and the occurrences of myalgia χ^2^ [(1, *N* = 49) = 7.29, *P* ˂ 0.05, Chi-square test] and Arthralgia χ^2^ [(1, *N* = 49) = 4.94, *P* ˂ 0.05, Chi-square test]. On the other side, there were no other associations between the presence of other comorbidities and the severity and the occurrence of symptoms during or post disease attacks (*P* > 0.05, Chi-square test).

Regarding clinical practice colchicine is the primary treatment modality used for FMF (89.6%). Other drugs are also used to lesser extent as corticosteroids (1.5%), non-steroidal anti-inflammatory agent like diclofenac sodium (1.5%). According to this study’s results, biological treatments were never mentioned to be used. Pertaining to treatment compliance, 90.3% of the study patients state that they are adherent to the treatment. Among the study subjects, only 88.5% take their medications regularly while 11.5% taking it during attack only.

## Discussion

FMF is one of the most monogenic auto-inflammatory diseases, inherited autosomal recessively. However, based on previous research heterozygote mutation and patients with only one mutation would get FMF disease expression.^[23]^

Based on our results, first degree related FMF parents would have 60% chance to get their children having FMF. Previous studies have supported this outcome, as a substantial body of research has indicated a higher incidence of MEFV gene mutation in siblings of first-degree relatives of FMF patients.^[24,25]^ Lachmann and coworkers studied FMF in a group of FMF patients, where 84% of them had at least two mutations and 12% had single mutation. According to their study group, 92% of first-degree relatives (parents or siblings) of these patients had mutation in MEFV gene.^[24]^

Patients diagnosed with FMF in Jordan were desperately upset from the premarital screening. Therefore, 82.1% of the study patients believe that is important to perform pre-marital testing for FMF mutations. This remarkable result would in part reflect the actual status of premarital screening in Jordan since the national premarital screening program in Jordan includes primarily thalassemia screening. Besides, this screening is performed at the Ministry of Health without paying consideration to any updates or effort to include other genetic mutations. Since the Arabic culture traditionally encourages first degree relatives’ marriage, we believe that our study would raise attention in the region to start including such important modifications. It is of high importance to initiate a nationwide initiative to explore the importance of premarital tests, along with the high risks associated with getting fake results. Unfortunately, good percentage of Jordanians tries their best to escape from such test although it is free and noninvasive one. This could be attributed to cultural beliefs or lack of awareness, rather than any other alternative reason.

After the Syrian crisis in 2011, the influx of Syrian refugees has placed significant pressures on the availability of existing services within hosting countries, specifically on health and education.^[26]^ Consequently, Syrian refugee participated in the increment of FMF cases in Jordan.^[27]^ Moreover, as the refugee camps are closed and performing scientific studies need complicated permission, studying such communities remains of high priority. It is noteworthy to mention that consanguineous marriage among such closed communities is very high. This is due to cultural beliefs, economic issues, and lack of awareness. Considering these facts and as these refugees are integrated in Jordanian community, studying the prevalence of FMF among them become of high importance.

FMF diagnosis according to our study was primarily performed through genetic blood testing, with some exceptions at which patients’ diagnosis was mainly based on symptoms. While other patients were suspected to have appendicitis or performed appendectomy then diagnosed with FMF. This misdiagnosis of FMF still present a challenge worldwide as past research reported such cases were patient were misdiagnosed with appendicitis or rheumatic fever before FMF with the most common misdiagnoses was appendicitis.^[28, 29, 30]^ Moreover, as found from our results surgical misdiagnosis of FMF patients is still present. This result is also had been investigated in many studies that concluded that acute abdominal pain suffered by FMF patients may lead to an unnecessary laparotomy before FMF diagnosis.^[31]^

Common clinical features of FMF indicate that recurrent episodes of abdominal pain and fever still the main distress suffered by FMF patients.^[14,32]^ Those two symptoms; fever and abdominal pain are also mostly suffered by the study participants beside arthralgia and myalgia just before and during the disease attack.

On the other hand, most of the study patients suffered from insomnia and general fatigue just after the disease attack. As they state that disease attacks are most of the time connected to a state of irritability, insomnia and fatigue. This result can be explained according to the published literature in two ways; one in part is related to the used treatment during the disease attack which is colchicine as one of the study connected colchicine therapy to major depressive state affect the patients upon medication administration.^[33]^ The other explanation could be related to the disease state is self as FMF attacks may be triggered due to change in the circadian rhythm or the attack itself my lead to emotional distress and sleep disturbances.^[34]^ Other published literature found a state of increased histamine levels in FMF patients that may lead to a state of insomnia.^[35]^ Our results need further investigation since few studies are published with regard to this issue.

Many systems are affected by FMF, including the renal, mus-culoskeletal, and gastrointestinal systems. Moreover cardiovascular involvements are still reported, it is essential to diagnose those complications as early as possible as some of them can lead to an increase in morbidities and/ or mortality.^[36]^ As shown by our study results, the participants suffer from chronic morbidities like the previously mentioned. Hypertension incidence was the highest among study participants compared to other diseases, and it was associated with an increase in the severity of arthralgia and myalgia. The association of FMF and cardiovascular disease was investigated in past research and attributed to the subclinical atherosclerosis and Deposition of amyloid.^[37]^ Research by Yalçinkaya *et al*. (2019) demonstrated a compelling link between hypertension and heightened occurrences of myalgia and arthralgia among FMF patients in Jordan. This correlation not only underscores the intricate pathophysiological mechanisms underlying FMF but also highlights the critical importance of comprehensive management strategies tailored to address both FMF-related symptoms and co-existing chronic conditions. As such, elucidating these associations not only enhances our understanding of FMF pathogenesis but also paves the way for more targeted and effective therapeutic interventions aimed at improving patient outcomes.^[38]^

Colchicine is the primary treatment of FMF as found in the current literature and clinical practice all over the world.^[39]^ This practice is still adopted in Jordan as we can found from our results that our patients were mainly on colchicine treatment either on a regular base or during FMF attacks.

## Conclusion

In conclusion, our study sheds light on the interplay between FMF and chronic diseases, particularly highlighting the association with hypertension and its impact on symptom severity among FMF patients in Jordan. Our findings assert the importance of premarital tests and shed light on the importance of incorporating FMF test as part of premarital testing. The issue of misdiagnosis remains a major challenge in Jordanian clinical practice. This emphasizes the urgency of instituting advanced training programs for health care providers.
